# Supplementary material for: Curated and harmonised transcriptomics datasets of interstitial lung diseases
Source: Data Brief. 2025 Oct 14;63:112139. doi: 10.1016/j.dib.2025.112139 (PMC12581653; doi:10.1016/j.dib.2025.112139)
Supplement: Supplementary file 1 [file mmc1.zip › Supplementary_material/DNA-microarray/GSE53845/GSE53845_eUTOPIA_Agilent_QC_Report_2023-12-15.tar.gz!/Users/OWNER/AppData/Local/Temp/RtmpAFKb7F/2023-12-15/hm.pdf]

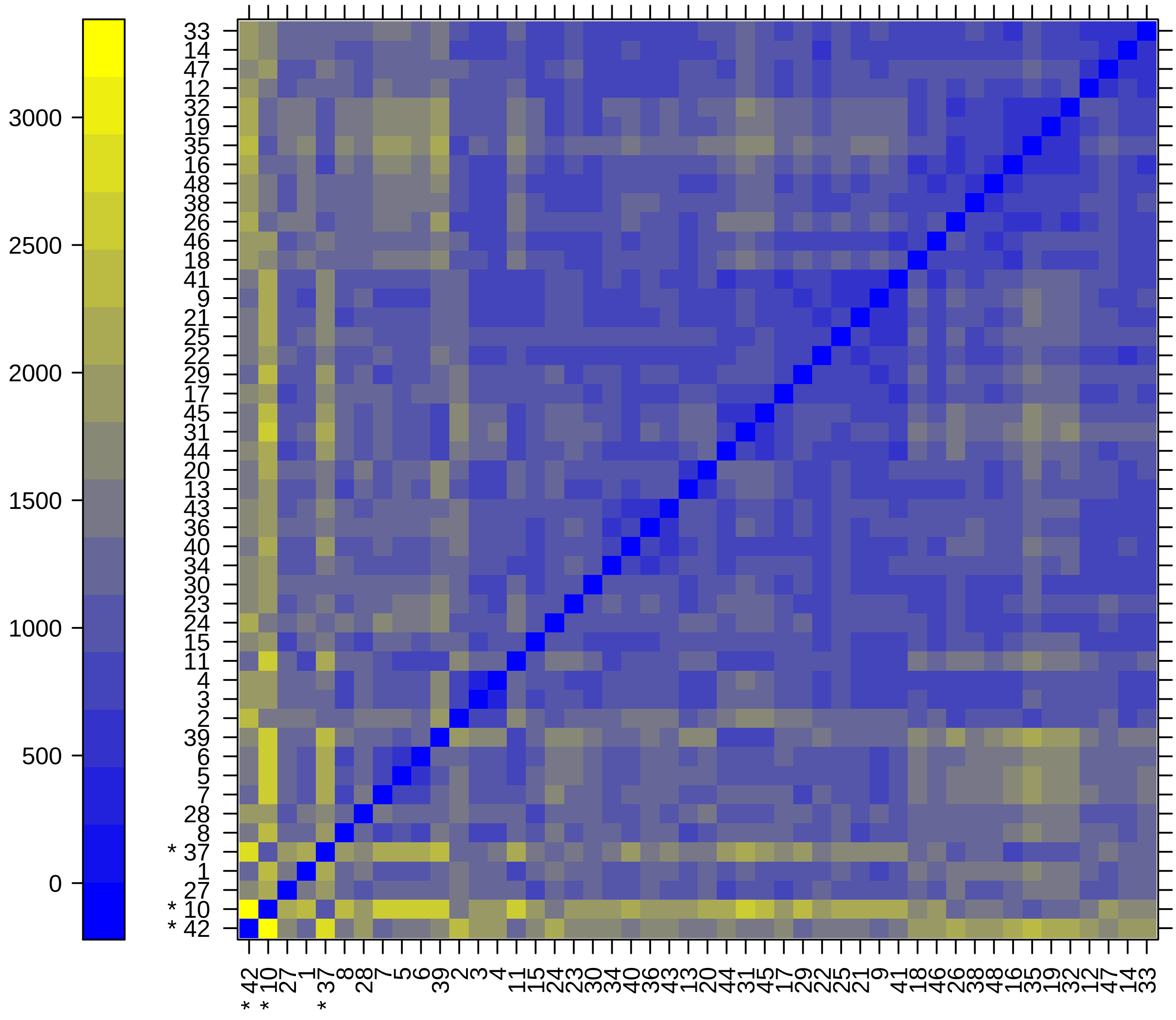
3000
2500
2000
1500
1000
500
0
33 14 47 12 32 19 35 16 48 38 26 46 18 41 9 21 25 22 29 17 45 31 44 20 13 43 36 40 34 30 23 24 15 11 4 3 2 39 6 5 7 28 8 * 37 1 27 * 10 * 42
* 42 * 10 27 1 * 37 8 28 7 5 6 39 2 3 4 11 15 24 23 30 34 40 36 43 13 20 44 31 45 17 29 22 25 21 9 41 18 46 26 38 48 16 35 19 32 12 47 14 33
